# Ecosystem Service-Based Deconstruction of Ancient Tree Values: Implications for Biodiversity Conservation and Socio-Ecological Management

**DOI:** 10.3390/plants15132064

**Published:** 2026-07-02

**Authors:** Yiwei Han, Zhenfan Liu, Lanbin Li, Zuxing Wei, Yue Pan, Ming Chen, Donghui Peng

**Affiliations:** 1 College of Landscape Architecture and Art, Fujian Agriculture and Forestry University, Fuzhou 350100, China; 62519052003@fafu.edu.cn (Y.H.); 62419052020@fafu.edu.cn (Z.L.);; 2 College of Architecture and Engineering, Fujian Water Conservancy and Electric Power Vocational College, Yong’an Campus, Sanming 366000, China

**Keywords:** ancient trees, large old trees, ecosystem services, bibliometric analysis, carbon storage

## Abstract

Ancient and large old trees hold significant ecological, cultural, and landscape importance. While numerous studies have investigated the ecosystem services these trees provide—such as carbon sequestration, air purification, and microclimate regulation—the findings remain dispersed and fragmented. To address this gap, the present study employs ecosystem service theory alongside a combination of bibliometric analysis and systematic review, examining 217 articles indexed in the Web of Science Core Collection. This approach offers a comprehensive synthesis of global research on ancient trees. The bibliometric results reveal a rapid expansion of research since 2015, with a particularly notable surge after 2019. Research focus has progressively shifted from traditional resource surveys and conservation management toward regulating services, emphasizing ecosystem functioning, carbon storage, microclimate regulation, and biodiversity conservation. Concurrently, methodological approaches have diversified, incorporating GIS-based spatial analysis, remote sensing techniques, and carbon storage modeling. Despite these methodological advancements, current research faces several challenges, including insufficient integration across spatial and temporal scales, limited long-term dynamic monitoring, and a weak linkage between ecological functions and sociocultural values. To enhance the protection and revitalization of ancient trees, future investigations should adopt multiscale and interdisciplinary frameworks that integrate ecological functions, landscape spatial dynamics, and sociocultural dimensions. Such approaches will facilitate the sustainable management of ancient trees globally and ensure the enduring provision of their ecosystem services.

## 1. Introduction

Ancient trees are biological legacies shaped by long-term natural processes and sustained human management, protection, and cultural appreciation. As distinctive ecological elements, they perform important ecosystem functions while embodying cultural and symbolic meanings. They record ecosystem succession and embody local belief systems, collective memory, and landscape identity, thereby serving as integrated carriers of natural and cultural heritage. Numerous studies show that ancient trees provide key ecosystem services in both urban and rural contexts, including carbon sequestration, microclimate regulation, soil conservation, habitat support, and cultural identity reinforcement [1,2,3].

In the context of climate change and rapid urbanization, ancient trees play a crucial role in maintaining ecological stability and enhancing human well-being. Compared with ordinary trees, they develop complex biological structures over long periods, resulting in unique ecosystem service mechanisms. These are reflected in habitat provision, microclimate regulation, and ecological process maintenance. Structurally, features such as large trunks, cavities, fissured bark, and decayed tissues provide stable microhabitats for diverse organisms—including insects, birds, mosses, lichens, and fungi—supporting higher biodiversity than younger forests [4,5]. Their complex canopy structure regulates radiation and airflow, contributing to local microclimate stability [6]. Additionally, deadwood from senescent stages supports decomposition and soil activity [7] and provides food resources for fauna [8], thereby linking supporting and regulating services. Beyond ecological roles, ancient trees are deeply embedded in human societies, representing historical memory, social identity, and cultural symbolism and indirectly promoting ecological conservation through public awareness and engagement [9,10].

However, with expanding urbanization, infrastructure development, and increasing extreme climate events, ancient tree habitats are shrinking, threatening their structural integrity and ecological functions. Current conservation strategies mainly rely on administrative management and physical protection, focusing on individual tree rescue and safety [11], but lack systematic, ecosystem service-based evaluation and dynamic planning. This “individual survival-oriented” approach fails to address the broader ecological and cultural roles of ancient trees within regional systems [12,13]. Moreover, by focusing primarily on the survival and safety of individual trees, existing approaches often overlook their broader roles in carbon storage, microclimate regulation, habitat connectivity, and cultural landscape formation, limiting their integration into climate adaptation and spatial planning strategies.

Under the goals of ecological civilization and carbon neutrality, the ecosystem services of ancient trees have become a growing research focus [14,15,16]. Although ancient trees exhibit more complex structures and cumulative ecological effects than ordinary trees, quantitative studies remain limited and often rely on models designed for general trees, lacking specificity. Meanwhile, increasing attention has been paid to their role in cultural landscapes and social networks. For example, Madera [17] emphasized that the loss of traditional shrublands represents both ecological and cultural decline. Studies in Japan have revealed links between ancient trees and community spaces through “shrine forest” social networks [18]. Research from China, South Korea, and Spain has further developed the concept of ancient tree cultural landscapes [19,20,21,22], while British scholars proposed the “ecosystem humanistic landscape,” highlighting integrated conservation approaches [23].

Despite the growing recognition of ancient trees as providers of multiple ecosystem services, it remains unclear whether current research has developed a balanced and integrated understanding of their ecological and socio-cultural values. Drawing on broader ecosystem service literature, several recurring challenges have been identified, including uneven attention among service categories, methodological heterogeneity, and limited integration of ecological and social dimensions. Accordingly, this review addresses the following research questions:

**RQ1:**  *Has research on ancient-tree ecosystem services been evenly distributed across provisioning, regulating, supporting, and cultural services?*

**RQ2:**  *To what extent have differences in data sources, assessment methods, and spatial scales affected the comparability and integration of ecosystem service evaluations?*

**RQ3:**  *How effectively have ecological, social, and governance dimensions been incorporated into existing assessments of ancient-tree ecosystem services?*

To address these gaps, it is necessary to systematically integrate ancient tree research within an ecosystem service framework. This study applies knowledge mapping methods using CiteSpace 6.4.R1 and VOSviewer 1.16 to analyze the knowledge structure and evolution of research on ancient tree ecosystem services. The objectives are:

(1) to identify the knowledge structure and evolutionary stages through co-occurrence and mapping analysis;
(2) to integrate regulating, supporting, and cultural services and construct a systematic conceptual model of ancient tree multifunctionality;
(3) to identify research hotspots and methodological trends, clarify gaps, and provide a theoretical basis for comprehensive assessment and collaborative governance.

## 2. Materials and Methods

Globally, there is no unified definition of ancient trees. In the United Kingdom, Natural England defines an “ancient tree” as one in an advanced life stage with distinct ecological and morphological traits [24,25]. In the United States, the “Champion Tree Program” evaluates tree significance using height, canopy spread, and trunk diameter [26]. Terms such as “heritage tree” [27] and “large old trees” [8] are also widely used. In China, protection policies since the 1980s define ancient trees as those over 100 years old, with a three-level classification: Level 1 (>500 years), Level 2 (300–500 years), and Level 3 (100–300 years). Overall, definitions have evolved from a single age-based criterion to a multidimensional framework incorporating morphology, ecological function, and cultural value, reflecting a shift from individual tree protection to integrated ecosystem and cultural landscape conservation.

Efforts to protect ancient trees in Europe date back to the late nineteenth century, exemplified by the British “Ancient Tree Preservation Movement,” which promoted ecological and cultural conservation through legislation and public engagement [28,29]. In the twenty-first century, the European Union incorporated ancient forest conservation into its “Biodiversity Strategy for 2030” [30], while countries such as France [31] and Italy [32] have integrated ancient trees into cultural landscape management. In the United States, forest and climate policies have strengthened protections for old-growth forests on federal lands [33]. Meanwhile, initiatives such as the EU “Ancient Tree Forum” support data sharing on distribution, health, and ecological value [34], and the UK “Ancient Tree Inventory” enhances spatial identification and prioritization of ancient-tree networks [35]. In addition, countries including Poland [10], Brazil [36], and China [37,38] increasingly adopt community-based conservation approaches that integrate ecological protection with cultural heritage transmission.

**Figure 1:**
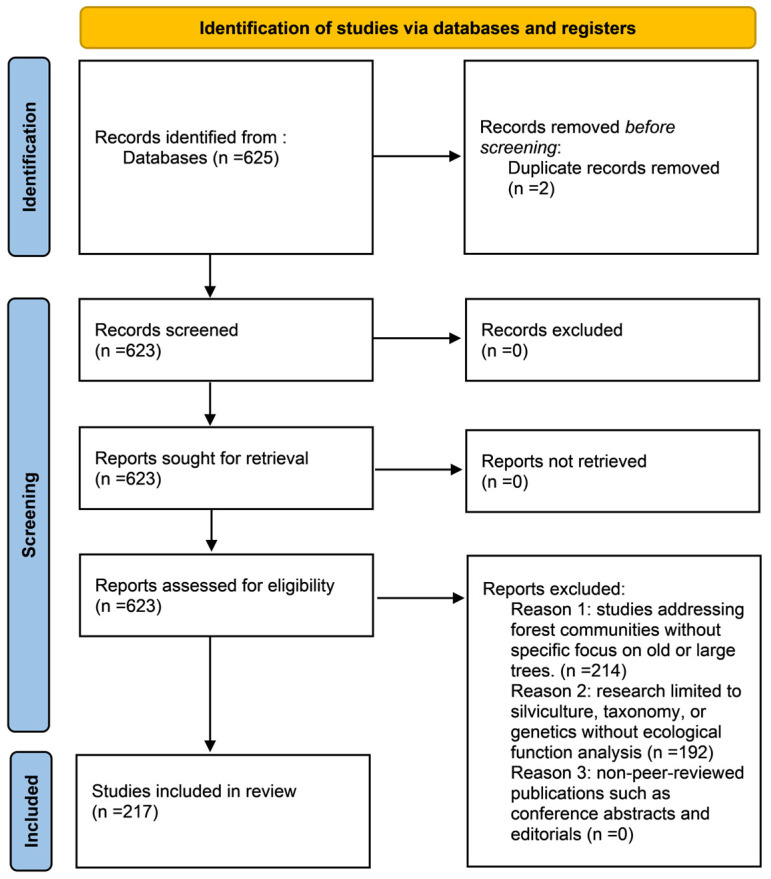
Systematic review process.

For the systematic review of ecosystem services provided by ancient trees, this study adheres to the PRISMA protocol for systematic reviews [39] (Figure 1). The process of the systematic evaluation is shown in Figure 1. This study followed Adriaanse [40] and selected the Web of Science Core Collection (WoSCC) as the primary database due to its comprehensive coverage and citation reliability. The search was completed on 11 February 2026, with document types limited to Articles and Review Articles, covering publications up to 31 December 2025.

The finalized search query was as follows:

TS = ((“ancient tree” OR “heritage tree” OR “old tree*” OR “old-growth tree” OR “large old tree” OR “veteran tree” OR “large-diameter tree” OR “mature tree” OR “large crown tree”) AND (“ecological function” OR “ecosystem function” OR “carbon sequestration” OR “carbon storage” OR “transpiration” OR “evapotranspiration” OR “microclimate regulation” OR “cooling effect” OR “air purification” OR “air quality” OR “particulate matter” OR “ecosystem services” OR PM2.5 OR PM10)).

To improve retrieval accuracy, multiple rounds of search optimization were conducted, yielding 625 initial records. Literature screening followed predefined criteria. Inclusion criteria were: (1) focus on ancient, heritage, or large-diameter trees; (2) examination of ecological functions or ecosystem service regulation; and (3) use of empirical data, modeling, or systematic review approaches.

Exclusion criteria included: (1) studies addressing forest communities without a specific focus on old or large trees; (2) research limited to silviculture, taxonomy, or genetics without ecological function analysis; and (3) non-peer-reviewed publications such as conference abstracts and editorials.

The lead reviewer extracted objective information from each full-text study into a standardized table, covering bibliographic details, research questions, study sites and subjects, data sources, data types, algorithms and software, analytical procedures, principal findings, and reported advantages and limitations. Other researchers independently checked an initial subset of entries to calibrate the extraction form and finalize variable names and definitions. After calibration, the full dataset was processed, and the remaining entries were verified against the original texts. A senior researcher coordinated consistency checks and resolved any disagreements through discussion.

**Figure 2:**
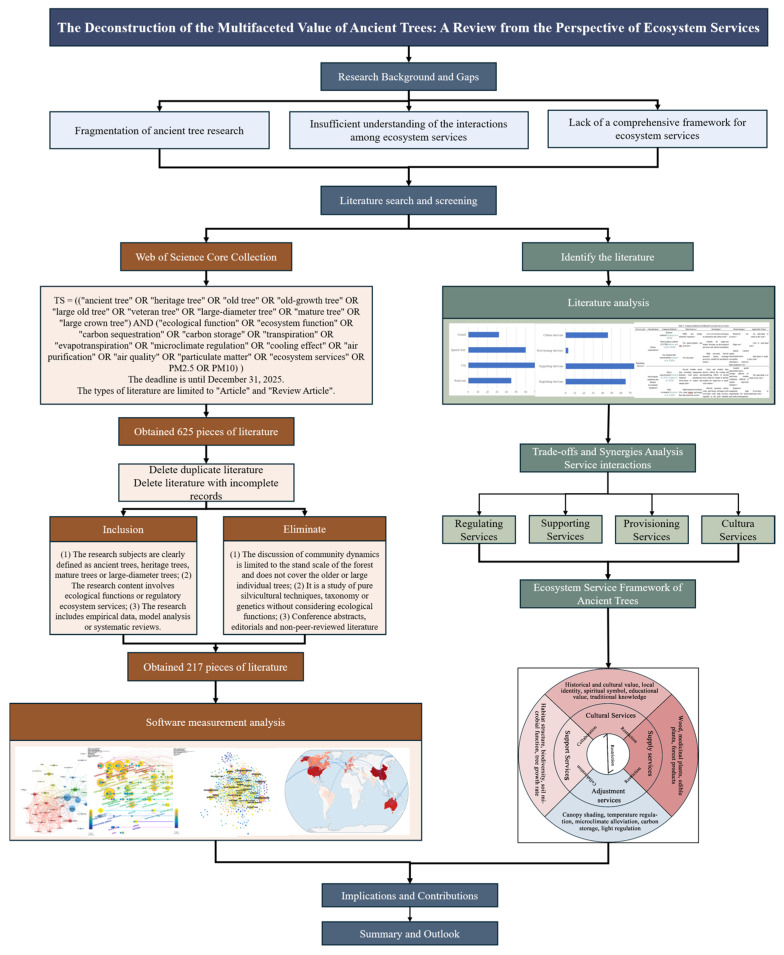
Technology Roadmap.

After removing duplicates and screening titles, abstracts, and full texts, 217 publications were retained for analysis (File S1). Bibliometric analysis was conducted using CiteSpace (version 6.4.R1), VOSviewer (version 1.6), and Microsoft Excel 2016, covering publication trends, collaboration networks, keyword co-occurrence, thematic evolution, and research hotspots (Figure 2).

## 3. Research Results and Discussion

### 3.1. Bibliometric

(1) Bibliometric Results

As of December 2025, a total of 217 publications on the ecosystem services of ancient trees were identified, including 198 research articles (91.3%), 12 review articles (5.5%), 2 editorial materials (0.9%), and 5 proceedings papers (2.3%). The dominance of journal articles indicates a strong emphasis on empirical and case-based research, while the limited number of reviews highlights the need for more systematic syntheses and comprehensive conceptual frameworks.

**Figure 3:**
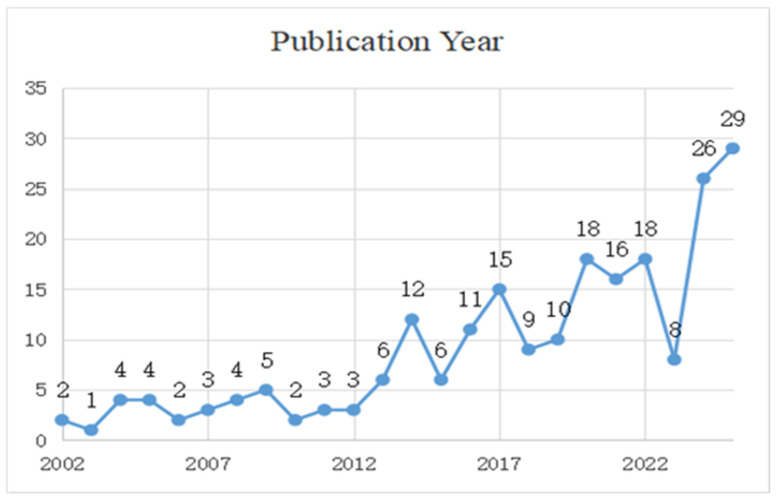
Annual Publication Trends.

Publication output remained low during the early stage (2002–2012), increased steadily after 2013, and grew rapidly in the most recent period (2019–2025), suggesting that this field has become an emerging research hotspot (Figure 3).

**Figure 4:**
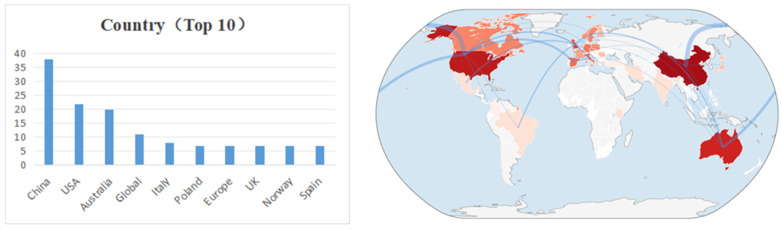
Number of Government-Issued Official Documents.

The national distribution of publications shows a clear concentration in countries with strong research capacity. China ranks first with 38 publications, followed by the United States (22) and Australia (20), while 11 studies adopt a global or cross-regional perspective (Figure 4). Spatially, research activity is clustered in East Asia, North America, Western Europe, and Oceania.

**Figure 5:**
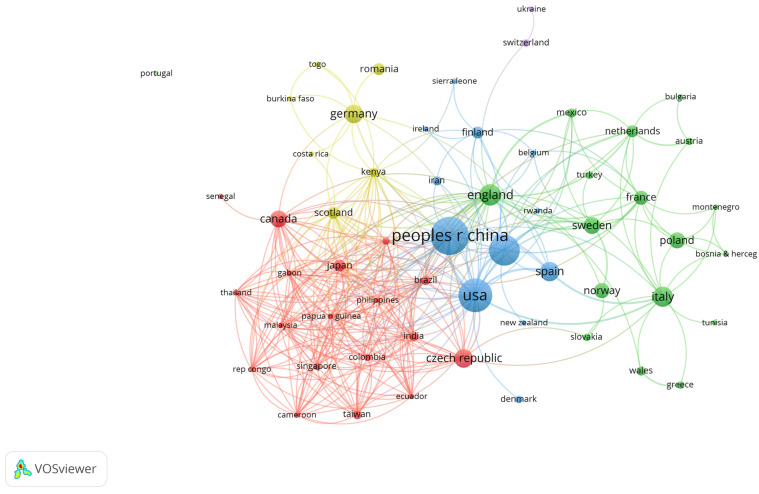
International Collaboration Network.

The international collaboration network further indicates that China, the United States, and Australia occupy central positions, with the highest node weights and link strengths (Figure 5). In contrast, countries in Africa and South America remain at the periphery, and overall collaboration intensity is relatively low. Overall, the global research pattern is dominated by China, the United States, and Australia, with additional contributions from several European countries.

**Figure 6:**
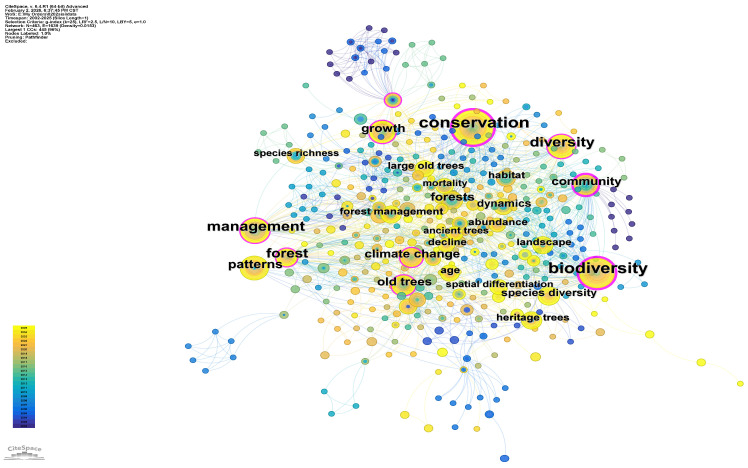

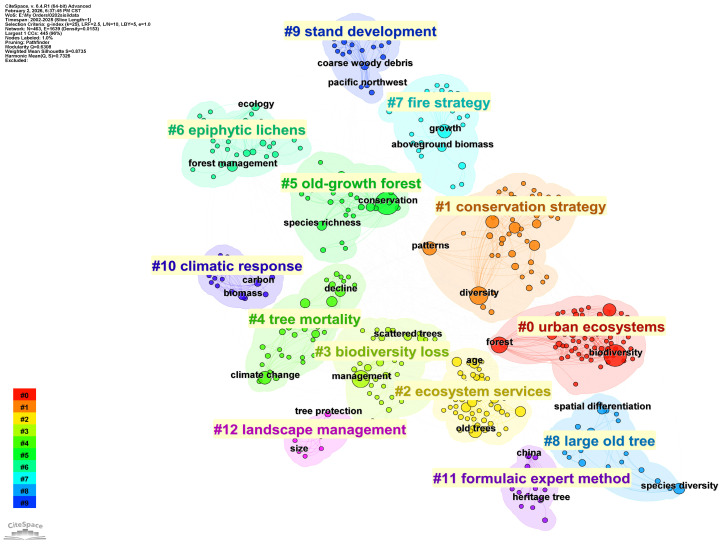
Keyword Co-occurrence and Clustering Analysis.

Keyword co-occurrence and clustering analyses reveal that ancient tree research has shifted from traditional forest ecology toward urban conservation, ecosystem services, and biodiversity protection under climate change (Figure 6). Major themes include conservation, biodiversity, management, urban ecosystems, ecosystem services, tree mortality, and old-growth forests, while smaller clusters reflect increasing specialization in disturbance ecology and management practices.

**Figure 7:**
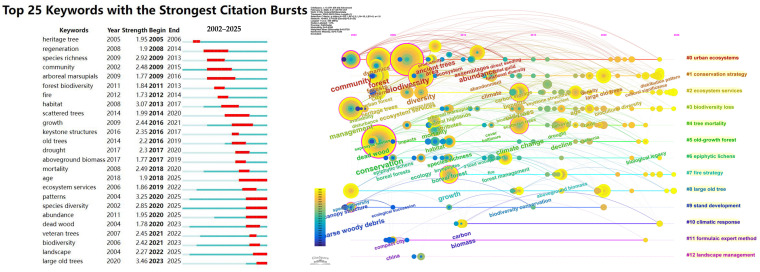
Keyword Burst Detection and Timeline Analysis.

Temporal and burst analyses further indicate evolving research priorities (Figure 7). Early studies (2002–2010) focused on community dynamics and forest management, whereas later research emphasized climate change, habitat dynamics, urban ecosystems, and ecosystem services. Since 2021, increasing attention has been given to large old trees, biodiversity conservation, and landscape-scale analysis. Overall, the field has developed into a multiscale framework integrating forest ecology, urban ecology, and landscape management.

(1) Bibliometric Trends and Functional Emphases

Bibliometric patterns reveal that current research priorities are closely associated with policy agendas related to carbon neutrality, climate adaptation, and urban resilience. Consistent with the systematic review results, regulating services dominate ancient tree studies, whereas provisioning and cultural services remain comparatively underrepresented. This imbalance suggests that ecosystem service assessments still primarily emphasize measurable ecological functions, while the interactions between ecological processes, cultural values, and governance mechanisms receive less attention.

Geographically, research is concentrated in Europe, East Asia, and other developed regions, while tropical and subtropical ancient tree systems remain insufficiently studied. From a methodological perspective, ecosystem service and carbon cycle models developed for temperate regions may not be directly applicable to tropical and subtropical ecosystems. Owing to their rapid carbon turnover and disproportionate contribution to the terrestrial carbon cycle under humid climatic conditions [41], tropical and subtropical forests differ substantially from temperate ecosystems in species composition, ecosystem structure, and carbon dynamics [42]. Consequently, applying models developed for temperate regions without regional recalibration may introduce considerable uncertainty into ecosystem service assessments. Expanding research efforts in underrepresented regions and incorporating region-specific assessment approaches will be essential for improving the global representativeness of ancient tree ecosystem service studies.

### 3.2. Overview of Research on the Ecosystem Services of Ancient Trees

#### 3.2.1. Research Area

A statistical analysis of 217 publications on ancient tree ecology (Table 1) shows that current research is mainly concentrated in urban environments and specific ecological regions, while rural landscapes remain underrepresented. Urban-related studies account for the largest proportion, exceeding one-third of the total, and primarily focus on ancient urban trees, street trees, and urban heritage forests. These studies emphasize growth characteristics, microclimatic regulation, and ecosystem service functions under urbanization pressures.

Research in specialized ecological zones, including nature reserves, primary forests, and mountainous or tropical ecosystems, also represents a substantial share of the literature. This body of work mainly examines large ancient trees in protected or natural forest systems, highlighting their importance for biodiversity maintenance, habitat structure, and ecosystem stability. In contrast, studies in rural contexts are relatively scarce and mainly address agroforestry systems and cultural landscapes. Only a small number of studies adopt global or multi-regional comparative perspectives, typically through literature reviews or cross-regional assessments of conservation strategies and ecological value.

Overall, the spatial distribution of research shows a dual focus on urban ecosystems and nature reserves, while rural ancient tree systems and cross-regional comparative studies remain limited. Given that rural areas constitute the primary habitat of many ancient trees, existing research tends to emphasize cultural services, indicating a need for further studies on their ecological functions and conservation strategies in rural landscapes.

#### 3.2.2. Types of Ecosystem Services

An analysis of 217 studies (Table 2) shows that current research on ancient trees mainly evaluates ecological value through four ecosystem service categories: regulating, supporting, provisioning, and cultural services. The classification follows a multi-label approach, meaning that a single publication may be assigned to multiple ecosystem service categories and indicators.; thus, the sum of publications across categories and indicator frequencies may exceed the total number of reviewed studies (n = 217).

Among these, supporting and regulating services dominate the literature. Supporting service studies focus on biodiversity maintenance and ecosystem stability, commonly using indicators such as species richness, Red List species presence, tree growth rates, and soil microbial functions. Regulating service research emphasizes microclimate regulation and ecological moderation, including canopy shading, temperature regulation, carbon sequestration, and light attenuation, highlighting the role of ancient trees in reducing thermal stress and enhancing ecosystem resilience.

Provisioning services have received relatively limited attention, mainly related to medicinal plants. They are usually found in agroforestry systems or nomadic or rural landscapes. Cultural service studies explore historical heritage, local identity, spiritual symbolism, and traditional ecological knowledge, emphasizing the socio-cultural importance of ancient trees alongside their ecological roles.

Many studies address multiple ecosystem service categories simultaneously. Supporting and regulating services are most frequently analyzed together, particularly in research linking biodiversity conservation with microclimate regulation and carbon storage. Supporting and cultural services are also commonly integrated, especially in traditional villages and cultural landscapes, where ecological functions and cultural identity are closely intertwined. Provisioning services are often examined in combination with other categories while rarely studied in isolation.

Despite this breadth, the literature still lacks a clear understanding of the mechanisms underlying ecosystem service delivery. Most studies focus on identifying or quantifying functions such as carbon storage, habitat provision, and cooling effects but pay less attention to the driving processes and causal pathways. In particular, interactions among tree structure, physiological processes, environmental conditions, and human disturbance remain fragmented and lack an integrated theoretical or modeling framework. Cross-scale mechanisms—from individual trees to landscapes—are also insufficiently synthesized. As a result, current knowledge remains largely descriptive, underscoring the need to shift toward integrated, multi-factor mechanistic studies that combine diverse datasets and modeling approaches to better explain how ancient tree ecosystem services are formed and regulated.

### 3.3. Literature Review

Within the framework of ecosystem services, ancient trees demonstrate unique contributions due to the intricate structures developed over extended periods of growth. This uniqueness is predominantly evident in the regulating, supporting, and cultural services associated with ancient tree ecosystems. Conversely, provisioning services have received comparatively limited attention in studies concerning ancient trees, primarily because these trees are subject to stringent protection regulations and their resource utilization is largely non-extractive. Only a limited number of investigations have explored provisioning aspects, such as fruit-bearing species or indirect forms of resource provision [43,44]. Consequently, the present review focuses on regulation, support, and cultural services and systematically synthesizes advancements in research and methodological approaches related to the ecosystem services of ancient trees.

#### 3.3.1. Research Methods for the Assessment of Ancient Tree Ecosystem Services

To improve the comparability and practical relevance of the methodological framework, this study provides a comprehensive summary of the principal methods employed in the existing literature (Table 3). Research on the ecosystem services provided by ancient trees has progressively expanded from focusing on individual trees to encompassing landscape and regional scales. Investigations into physiological processes predominantly remain at the individual-tree level, whereas remote sensing and modeling techniques facilitate integrated assessments across multiple scales. This progression reflects a methodological shift in ancient tree research from isolated measurements to multiscale integrated simulations.

In studies addressing regulating services, model-based analyses and digital scanning techniques are predominantly utilized. Frequently used methods include species distribution models such as MaxEnt, spatial analysis, remote sensing, spectral analysis, and canopy structure scanning. These approaches, however, demand high-quality data and precise spatial resolution, and some still necessitate validation through field observations. Konôpka et al. [45] emphasized that tree age is a critical predictor of biomass and carbon stock estimates and that the conversion of biomass into litter and deadwood constitutes a significant process in forest carbon dynamics. Among the methodologies employed to simulate tree growth and carbon assimilation at physiological and morphological levels are the Functional–Structural Plant Model (FSPM) [46] and assimilation-based approaches [64].

Methodologically, research on the cooling effects of ancient trees primarily employs two approaches. The first involves direct measurements through on-site microclimate monitoring, such as deploying temperature and humidity sensors at approximately 1.5 m height and estimating cooling and humidifying effects by comparing tree-covered areas with adjacent bare ground serving as controls [48,49]. The second approach utilizes energy and water balance models that simulate transpiration processes, characterizing vegetation’s role in energy partitioning via evapotranspiration (ET) and the transpiration-to-evapotranspiration ratio (T/ET) [65,66]. Related techniques include eddy covariance [50], isotopic tracers [51], and model-based simulation [52]. Recent advancements incorporating three-dimensional canopy structure analysis and remote sensing technologies have further elucidated the significant regulatory influence of canopy complexity on transpiration fluxes [67].

Research concerning supporting ecosystem services predominantly employs conventional ecological survey methodologies. Recently, however, three-dimensional structural modeling approaches have been incorporated, including quantitative structure models (QSM) and mobile or terrestrial laser scanning techniques [63]. These approaches typically use data such as species distribution, tree age, diameter at breast height, and species monitoring records to evaluate the contributions of ancient trees to biodiversity maintenance, habitat provision, and the preservation of forest structural stability [61]. Compared to investigations of other ecosystem service categories, these methods offer a more precise representation of the ecological structural attributes of ancient trees. Nonetheless, the survey procedures are often labor-intensive, and the processes of data acquisition and analysis are comparatively complex.

The methodologies employed to assess the ecosystem services provided by ancient trees exhibit considerable diversity. In particular, studies focusing on regulating and provisioning services have increasingly adopted technologies such as remote sensing, three-dimensional laser scanning, and spatial modeling. Future research endeavors should aim to systematically integrate ecological, social, and cultural data to establish a more holistic scientific foundation for the conservation and management of ancient trees.

#### 3.3.2. Supporting Ecosystem Services

Ancient trees, characterized by their structural complexity and exceptional longevity, serve as critical habitats for a diverse array of plant and animal species [2,3]. Even post-mortem, the standing dead trees (snags) and fallen logs they leave behind continue to provide ecological niches for extended periods, often spanning several decades [7]. Consequently, ecosystems containing ancient trees generally exhibit elevated levels of biodiversity [4] and support the persistence of numerous rare or specialized taxa [68]. Beiler et al. [69] demonstrated that large, mature trees facilitate connectivity among subterranean root networks via fungal symbionts, thereby forming intricate nested structures and fulfilling a “mother tree” role in forest regeneration. Empirical studies further reveal that multiple ant communities, vascular plants, and moss species frequently coexist within ancient trees and their associated deadwood structures. In contrast, such species are comparatively scarce in forests dominated by younger stands.

In addition, tree age and larger stem diameters are considered important factors influencing ground lichen richness. Ancient trees often serve as the sole host for certain obligate lichens (such as *Letharia vulpina*) [15,70]. However, there is still some controversy regarding the impact of ancient forests on biodiversity. Some studies have found that ancient trees may reduce the complexity of the local soil microbial network by altering resource allocation and competition structure [71], or may lead to community assemblages dominated by generalist species [72]. These findings imply that the impact of ancient trees on biodiversity is highly context-dependent, necessitating a comprehensive interpretation of underlying mechanisms that accounts for spatial scale, disturbance history, and broader ecological context.

#### 3.3.3. Regulating Ecosystem Services

(1) Carbon Sequestration by Ancient Trees

Previous research consistently indicates that the conservation of mature and old-growth forests is crucial not only for sustaining the carbon sequestration capacity of terrestrial ecosystems but also for supporting a range of ecosystem services, such as biodiversity preservation and microclimate regulation [73]. Ancient trees, as structurally intricate elements within forest ecosystems, are characterized by substantial trunk girth, extensive canopy development, and elevated biomass density. The carbon storage capacity of individual ancient trees markedly exceeds that of typical trees. Within mature and primary forests, large mature and old trees account for approximately 41% to 84% of the total carbon stored in forest biomass [74,75]. Furthermore, the annual carbon accumulation rates of these ancient trees surpass those of medium- and small-sized trees [76,77,78], underscoring their indispensable role in maintaining long-term carbon sequestration stability. Comparative analyses across tree species have revealed significant interspecific variability in carbon sequestration potential [79]. Among the metrics employed, the QCO_2_ index, which quantifies the carbon sequestration capacity of individual trees, is considered a more accurate indicator of the ecological functionality of individual plants and urban green spaces than the wCO_2_ index.

Empirical investigations at the individual tree level have corroborated these findings. For instance, a study conducted by Ou Yang Cuiyu et al. [74] In Xiangshan, Beijing, ancient trees were found to have carbon storage values up to 13,178.32 kg, with trunk diameter, canopy volume, and carbon storage exhibiting significant linear correlations. Nonetheless, the majority of extant studies use static or simplified modeling approaches to examine the relationship between stand age and annual carbon sequestration, thereby limiting comprehensive understanding of carbon dynamics in ancient trees across various successional stages. Consequently, there is a pressing need to integrate long-term monitoring data with sophisticated modeling techniques to enable a more nuanced empirical examination of the successional processes that govern carbon storage in ancient trees.

(2) Microclimate Regulation and Thermal Environment Mitigation by Ancient Trees

The regulation of microclimates by ancient trees is one of the most practically significant ecosystem services in both urban and rural settings [80]. Their extensively developed canopies and elevated leaf area indices modulate radiation balance and energy exchange through mechanisms such as shading and transpiration, thereby mitigating the urban heat island effect and enhancing human thermal comfort [6,14]. Nonetheless, certain studies have reported that with increasing tree age, stomatal density and conductance tend to decline, accompanied by reductions in net photosynthetic and transpiration rates [81]. Empirical evidence indicates that *Pinus koraiensis* trees around 210 years old exhibit relatively high stomatal density [82], with stomatal row number and density increasing up to this age before subsequently decreasing. This pattern suggests that the cooling capacity of ancient trees does not increase linearly with age but is influenced by the interplay of tree age, physiological status, soil moisture availability, and environmental stressors. The precise mechanisms underlying these dynamics remain to be systematically quantified. Their cooling effects are influenced by canopy structural complexity and spatial configuration, which regulate transpiration and energy exchange processes [83,84], and are further shaped by surrounding urban form and meteorological conditions [85,86,87].

In summary, extant literature underscores the potential benefits of ancient trees in modulating thermal environments through shading, transpiration, and spatial structural complexity. However, a notable gap remains in the systematic integration of physiological traits, structural intricacies, and environmental adaptability in aged trees. Future investigations should adopt a multiscale coupling framework that integrates individual physiological characteristics, canopy structure, and urban form and develop quantitative models of microclimate regulation tailored to ancient trees. Such approaches are essential for a more accurate evaluation of their contributions to ecosystem service provision.

(3) Air Purification Function of Ancient Trees

The air-purification role of urban trees and forests is widely recognized as a critical ecosystem service [88]. Compared with medium- and small-sized trees, ancient trees with expansive canopies generally exhibit superior performance in total pollutant removal and annual transpiration rates. Consequently, urban forestry management prioritizes the preservation of large trees as a key strategy to optimize regulating ecosystem services [57]. The capacity of trees to remove atmospheric pollutants is determined by a combination of their biological traits, canopy architecture, individual size, and external environmental conditions [89].

At the microscopic level, pollutant removal is influenced by the structural characteristics of leaves and bark, which regulate the deposition of particulate matter and the adsorption of gaseous pollutants [90]. Leaf surface morphology and chemical properties are widely recognized as key determinants of dust retention efficiency [89,91]. Modeling studies further suggest that conifer-specific traits and canopy structural complexity enhance PM_2.5_ removal efficiency by increasing surface roughness and deposition area. For example, an individual *Picea pungens* was estimated to remove up to 1.79 kg of PM_2.5_ during the 2019 in-leaf season [92].

#### 3.3.4. Ecosystem Cultural Services

Beyond their regulatory and support functions, ancient trees also fulfill significant cultural roles. As products of prolonged interactions between natural environments and human societies, ancient trees are not merely long-lived components within ecosystems but also serve as vital repositories of historical memory, local identity, and spiritual symbolism. They occupy a central position within religious beliefs, traditional customs, and cultural landscape frameworks [93,94]. Numerous studies have demonstrated that ancient trees substantially enhance residents’ attachment to their locality and cultural identity by augmenting the aesthetic value of landscapes and reinforcing the symbolic and spiritual significance of places. These effects positively influence psychological well-being and social cohesion [9,10]. In urban contexts, tree-planting patterns, shaped by diverse cultural backgrounds, often mirror the historical trajectories and environmental transformations of cities, rendering urban green infrastructure an important archive of socio-ecological evolution [13]. Through this process, trees increasingly serve as “natural substitutes and symbols” in urban environments [95].

From the perspective of the “human–land relationship,” scholars have further emphasized that ancient trees represent not only ecological and cultural heritage resources but also tangible embodiments of social emotions and collective memory. In traditional spatial settings, ancient trees frequently serve as carriers of local memory, evoking emotional connections and fostering a sense of belonging among residents to historical places [96]. Research by Ingles highlights that the cultural identity and local sense of belonging among inhabitants of the Yucatan region are intimately linked to the forest and ancient tree landscapes [97], suggesting that ancient trees serve as landmarks in physical spaces while simultaneously reinforcing community identity at a psychological level.

The species diversity of ancient trees is shaped by historical planting preferences, clan cultures, and feng shui principles [35], with different species fulfilling distinct social functions and symbolic roles [98]. Global literature often references species such as *Quercus spp., Picea abies*, and *Olea europaea.* Ancient olive trees, in particular, not only bear witness to the history of olive cultivation but also exemplify environmental resilience [99,100]. Many individual olive trees, designated as “ancient olive trees,” serve as living testimonies to regional histories of olive cultivation and demonstrate the species’ remarkable endurance and vitality under challenging environmental conditions [101]. In China, northern regions are characterized by locust and cypress trees [102,103], whereas southern areas are dominated by camphor and ginkgo trees [104,105]. This spatial differentiation reflects not only ecological adaptation mechanisms but also the outcomes of long-standing socio-cultural preferences.

At the levels of institutional and social engagement, certain countries have begun incorporating ancient tree conservation into public co-management frameworks. For instance, the United Kingdom maintains an ancient tree registry through an online supervisory agreement, documenting approximately 190,000 ancient trees within cultural landscapes and promoting community involvement in their monitoring and updating processes [106]. Conversely, China’s model of social participation remains in its nascent phase; however, several cities have enhanced public awareness of tree protection through strategies such as “adoption, promotion, and education.” Ancient trees serve not only as repositories of historical memory but have increasingly become emblematic of collective cultural identity within communities, exerting a profound impact on the social fabric and everyday life of villages [107].

Although cultural ecosystem services are challenging to quantify with conventional ecological metrics, recent scholarship has begun to employ social surveys, landscape perception analyses, multi-criteria evaluation techniques, and spatial analytical tools to assess the cultural significance of ancient trees systematically. This emerging research trajectory reflects a paradigm shift from an “ecological function-oriented” perspective—primarily focused on biological and physical attributes—toward a “social–ecological integrated” approach that incorporates social dimensions. Such an integrative framework offers a more comprehensive and multidimensional theoretical basis for the conservation and management of ancient trees.

A comprehensive bibliometric analysis and systematic review reveal three prominent structural characteristics in the study of ancient tree ecosystem services. First, the research emphasis has shifted from traditional conservation and management concerns to climate regulation and carbon sequestration. Second, methodological approaches have advanced from reliance on single-indicator assessments to the integration of multi-source data and coupled modeling techniques. Third, investigations into ecological functions considerably outpace those addressing cultural and social dimensions. These structural patterns not only underscore the influence of global climate policy agendas but also highlight substantial opportunities to enhance theoretical synthesis and interdisciplinary collaboration in ancient tree research.

#### 3.3.5. Trade-Offs and Synergies Among Ecosystem Services

As research on ancient trees has increasingly been incorporated into the ecosystem services framework, interactions among regulating, supporting, and cultural services have become an important research focus. Ancient trees simultaneously provide carbon sequestration, habitat provision, and cultural benefits [9,10], but these services do not always increase synchronously.

Trade-offs may arise where cultural utilization, public access, safety management, or urban development constrain ecological functions. Ancient trees with high cultural significance are often located in temples, public squares, or historic settlements, where soil compaction, habitat simplification, and intensive management may weaken carbon sequestration, microclimate regulation, and biodiversity-supporting functions [8,108]. Likewise, insufficient consideration of ecosystem service values in land-use planning may reduce their contribution to ecological network connectivity [109,110].

Conversely, cultural services can generate important synergies. Cultural attachment, local identity, and community participation often strengthen public support for conservation and enhance the long-term stability of ecosystem service provision [10,111]. Recent advances in remote sensing, terrestrial laser scanning, digital twins, and ecosystem modeling have improved the quantification of multiple ecosystem services and provided new opportunities for evaluating trade-offs and synergies at individual-tree and landscape scales [74,112,113,114,115,116,117,118,119,120,121]. Integrating ecosystem service assessments into spatial planning, heritage conservation, and community-based governance would facilitate a transition from single-objective management toward multifunctional conservation, thereby improving the resilience of ancient tree socio-ecological systems.

**Figure 8:**
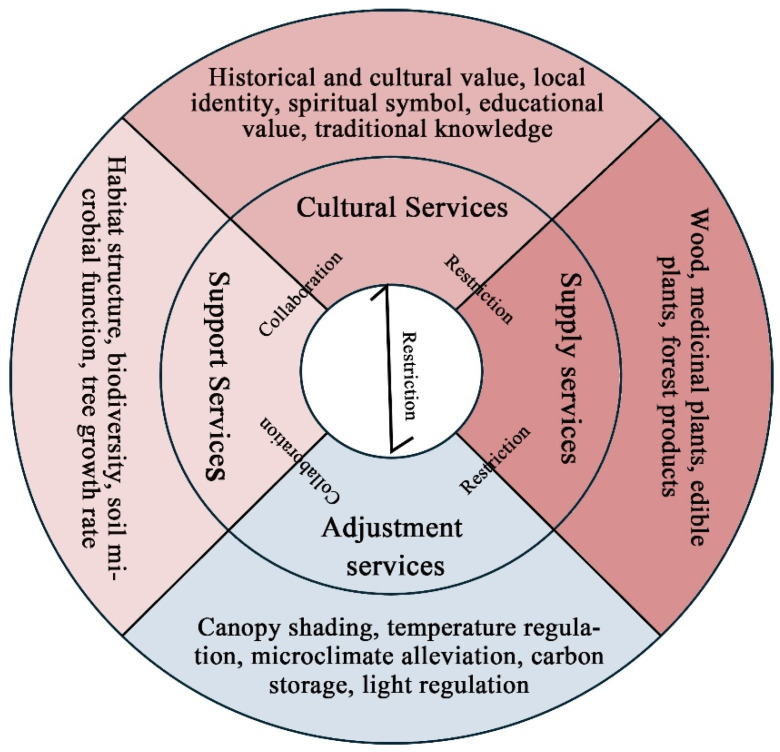
Relationships among ecosystem services.

These findings suggest that ancient tree conservation should move beyond single-objective management toward a multifunctional ecosystem service framework. Integrating ecosystem service assessments into spatial planning, heritage conservation, and community-based governance would help balance ecological functions and cultural values while improving the long-term resilience of ancient tree socio-ecological systems (Figure 8).

### 3.4. Protection and Management of Ancient Trees

#### 3.4.1. Concepts and Governance Frameworks for Ancient Tree Conservation

Over recent decades, the paradigm of ancient tree conservation has evolved from a predominantly biological protection focus to a more holistic framework that integrates both ecological and cultural dimensions. Initial research primarily focused on the physiological status and survival prospects of individual ancient trees, with conservation efforts largely oriented toward rescue interventions, including tree restoration, health maintenance, and lifespan extension [11]. However, with advances in ecosystem management principles, ancient trees have increasingly been recognized as critical components for sustaining ecosystem structure and cultural landscapes. Consequently, conservation objectives have broadened from safeguarding individual specimens to encompassing the integrated preservation of ecological and cultural values at the landscape level [12,13].

At the international scale, Europe pioneered a systematic approach to ancient tree conservation relatively early. The “Ancient Tree Preservation Movement,” which emerged in the United Kingdom in the late nineteenth century, is widely regarded as a foundational milestone in modern ancient tree conservation. This movement underscored the dual protection of ecological and cultural values through legislative measures and public education initiatives [28,29]. More recently, ancient tree conservation has been progressively embedded within global biodiversity policy frameworks. For instance, the European Union’s “Biodiversity Strategy for 2030” advocates enhanced, systematic protection of old-growth and primary forests [30]. In several European nations, ancient trees have been incorporated into cultural landscape heritage systems and are managed under protection regimes comparable to those applied to historic architectural structures [31,32]. Furthermore, organizations such as the United Kingdom’s “Ancient Tree Forum” have developed open-access database platforms that continuously update and disseminate information on the distribution, health status, and ecological significance of ancient trees [34]. This development reflects a transition toward institutionalized and data-driven governance models in ancient tree conservation.

Concurrently, public engagement has emerged as a vital complementary mechanism in the conservation of ancient trees. For example, various municipalities have promoted public awareness through activities such as tree-identification programs, scientific outreach, and cultural events, thereby fostering a sense of communal responsibility for protecting ancient trees. In traditional villages, ancient trees often embody local historical memory and cultural symbolism, exerting considerable influence on community identity and social cohesion [96,107,122]. Empirical studies have demonstrated a significant positive correlation between residents’ place attachment and their willingness to engage in ancient tree conservation [123], highlighting the critical role of socio-psychological factors in the governance of ancient tree preservation.

#### 3.4.2. Ancient Tree Health Assessment and Management Technologies

In contrast to the broader domain of conservation, the management of ancient trees is more narrowly focused on implementing practical conservation strategies and systematically monitoring tree health. The physiological condition of ancient trees is a critical determinant of their ecological roles and longevity. Consequently, health assessment and risk diagnosis have emerged as essential technical underpinnings for the effective management of these trees. Recent scholarly efforts have progressively advanced a comprehensive management framework that integrates field assessment, nondestructive testing, and intelligent monitoring technologies.

Regarding field assessment, traditional approaches primarily rely on morphological indicators—such as crown density, leaf discoloration, the proportion of dead branches, trunk inclination, and the presence of pathogens or pests—to assess the health status of ancient trees. To enhance the scientific robustness of these evaluations, researchers have developed quantitative health assessment systems, exemplified by the Tree Vitality Index, which synthesizes physiological parameters including leaf nitrogen content, sap flow rate, and trunk moisture content to provide a holistic appraisal of tree vitality [112]. Additionally, methodologies such as the Paine system, Visual Tree Assessment (VTA), and Forest Health Monitoring (FHM) establish multidimensional frameworks for health evaluation based on variables like crown vitality, trunk decay, and root exposure. [124,125,126,127]. Within the Chinese context, scholars have further incorporated multi-criteria evaluation techniques—such as the Analytic Hierarchy Process (AHP), Principal Component Analysis (PCA), and fuzzy comprehensive evaluation—to construct regional-scale health assessment models tailored to ancient trees [128,129]. More recently, multivariate statistical approaches, including Structural Equation Modeling (SEM), have been employed to elucidate causal relationships among tree structural attributes, environmental factors, and health indicators, thereby enhancing the explanatory and predictive capabilities of health assessment models [130,131].

Nondestructive diagnostic methodologies are vital tools for assessing the health of ancient trees, owing to their ability to obtain internal structural information without causing damage. Commonly utilized techniques encompass stress-wave detection, ultrasonic testing, resistance tomography, and infrared thermography [132,133]. Stress-wave tomography, for instance, detects decayed regions within the trunk by measuring variations in wave velocity, thereby accurately quantifying the extent of internal cavities. Resistance tomography leverages resistivity differences in wood to assess xylem moisture content and the extent of decay. Earlier diagnostic methods, such as increment-boring sampling and X-ray imaging, have also been employed [134,135,136,137]; however, their practical application is constrained by cumbersome equipment and operational complexity. In contrast, resistance meters are favored for their portability, ease of use, and cost-effectiveness, rendering them prevalent in field investigations and dendrochronological data collection [113]. By integrating resistance meter data with increment-boring and other nondestructive techniques, researchers can conduct detailed analyses of tree-ring structural characteristics and investigate the influence of climatic variables on tree growth dynamics.

With advances in information technology, the monitoring of ancient trees has become increasingly sophisticated. The deployment of environmental and physiological sensors on tree trunks and within root zones facilitates real-time measurement of critical parameters, including temperature, humidity, wind velocity, trunk strain, and photosynthetic rate, thereby enabling continuous, dynamic, long-term assessment of ancient tree health [138]. Additionally, aerial imaging via drones, combined with LiDAR (Light Detection and Ranging) technology, enables the generation of three-dimensional models of ancient trees, allowing precise monitoring of tree structure and canopy dynamics. Building upon these data, image recognition methodologies employing deep learning algorithms, such as convolutional neural networks (CNNs), can automatically detect features indicative of health issues, including disease symptoms, trunk fissures, and leaf deterioration. This integration markedly enhances the efficiency and accuracy of ancient tree monitoring and management practices.

In summary, contemporary technologies for ancient tree management are progressively shifting from reliance on traditional experiential judgment toward data-driven, refined approaches. At the governance level, conservation of ancient trees is increasingly shifting toward multi-actor and cross-sectoral coordination. By synthesizing diverse data sources with intelligent monitoring techniques, these advancements provide critical technical support for the sustained conservation of ancient trees and the early detection of risks to them. Nevertheless, institutional fragmentation persists among forestry management, urban planning, and cultural heritage protection, leading to inconsistent implementation of ecosystem service-based conservation strategies [107,111].

To address these gaps, a multi-level policy framework is urgently needed:

(1) Spatial planning integration: Ancient trees should be formally incorporated into territorial spatial planning systems as nature-based infrastructure and ecological assets with legally recognized ecosystem service values, rather than being treated solely as heritage objects.
(2) Cross-sector governance coordination: Integrated governance mechanisms linking ecological protection, urban development, and cultural heritage conservation should be established, ensuring that ecosystem service evaluation becomes a mandatory component of planning approval and land-use decision-making.
(3) Digital and intelligent conservation systems: The application of AI, IoT, and digital twin technologies should be promoted not only for monitoring but also for predictive management, early warning of risks, and scenario simulation to support adaptive decision-making.
(4) Ecosystem service-based policy instruments: Standardized valuation and accounting systems should be developed for regulating cultural services of ancient trees to enable their inclusion in ecological compensation, urban green infrastructure investment, and carbon neutrality strategies.

Overall, these measures are essential to shift ancient tree conservation from a passive protection paradigm toward an active, quantifiable, and governance-integrated ecosystem service management framework.

## 4. Research Limitations and Future Prospects

Ancient trees, as important components of biological heritage shaped by long-term ecosystem evolution, play a vital role in maintaining ecosystem stability and delivering multiple ecosystem services. Research in this field has gradually shifted from traditional forest ecology and protected area studies toward quantitative assessment and model-based approaches, reflecting a broader transition from descriptive ecology to functional and scenario-oriented analyses.

Current studies on ancient trees have developed along four interrelated dimensions: ecological function evaluation, health monitoring, cultural interpretation, and social governance. Together, these dimensions highlight the increasing integration of ecological, social, and technological perspectives within the ecosystem services framework. Despite notable progress in quantitative methods and analytical tools, significant limitations remain, particularly in terms of data integration, cross-scale coupling, and the comprehensive linkage between ecological processes and socio-cultural values.

(1) There remains a lack of comprehensive integration between ecological and social mechanisms. Current studies predominantly emphasize either natural processes or cultural perceptions, without developing a holistic model that systematically elucidates the interrelations among ecosystem services, place attachment, and conservation behaviors.
(2) There is an inadequate linkage between planning and governance frameworks. Ancient trees are frequently excluded from effective incorporation into territorial spatial planning and broader ecosystem service provision strategies. This gap underscores the urgent necessity to establish cross-scale, multisectoral collaborative management mechanisms.
(3) The degree of digitalization and intelligent management applied to ancient tree conservation is still limited. Future efforts should focus on the enhanced integration of digital twin technologies, the Internet of Things, and artificial intelligence to facilitate dynamic assessments of ecosystem services, early risk detection, and informed decision-making. Such advancements would support the transition from traditional empirical management approaches toward more scientific and intelligent governance models.

## 5. Conclusions

This study integrates bibliometric analysis and systematic review to examine 217 publications on ancient and large old trees (2002–2025), revealing key research trends, functional characteristics, and methodological developments in ecosystem service studies.

(1) Research Trends and Thematic Shift

Publication output has increased steadily, with research focus shifting from early-stage resource inventory and conservation management to ecosystem function assessment and climate-related studies after 2015, followed by rapid expansion in recent years.

(2) Geographic Distribution and Research Imbalance

The global research network exhibits a core–periphery structure, dominated by China, the United States, and Australia, alongside strong European collaboration. However, studies are largely concentrated in temperate regions, with limited representation of tropical and subtropical systems, indicating significant spatial bias.

(3) Ecosystem Service Contributions of Ancient Trees

Ancient trees provide substantial regulating and supporting services, particularly in carbon sequestration, microclimate regulation, and habitat provision. Their structural complexity enables disproportionately high ecological contributions at the individual scale, highlighting their irreplaceable role in ecosystem functioning.

(4) Trade-offs and Synergies Among Ecosystem Services

Ecosystem services exhibit clear coupling relationships: regulating services form the biophysical foundation, supporting services sustain ecological processes, and cultural services influence service delivery through social feedback. Trade-offs emerge under high human disturbance, while synergies are facilitated by community engagement and conservation awareness.

(5) Methodological Advances and Limitations

Research methods are evolving from single-indicator assessments toward integrated, multisource modeling approaches. Despite advances in remote sensing, terrestrial laser scanning, and ecological modeling, robust frameworks for multiservice coupling and long-term dynamic monitoring remain limited, and emerging technologies such as digital twins remain underexplored.

Despite notable progress in publication output and methodological innovation, several critical limitations persist in the field. First, the geographic and species representation within the existing literature is uneven, with particularly sparse data from developing countries. Second, research on carbon sequestration and microclimate regulation predominantly employs static estimation methods and lacks long-term dynamic monitoring. Third, an integrated framework that connects ecological functions, cultural values, and policy systems has yet to be established. Future research must prioritize developing multiscale monitoring systems, promote the standardization and regional validation of carbon cycle and ecosystem service models, and integrate ecological functions, cultural identity, and governance mechanisms from a social–ecological perspective. Such efforts are essential to strengthen the scientific foundation for the conservation and adaptive management of ancient trees.

In the context of global climate change and escalating urban heat stress, ancient trees represent not only elements of ecological heritage but also critical landscape components with enduring regulatory capacity and cultural significance. Therefore, their conservation and functional optimization carry substantial ecological and societal implications.

## Figures and Tables

**Table 1:** Number of Study Area Types.

Study Area Type	Notes/Representative Cases	Number of Publications
Rural areas	Agricultural, Forestry, and Pastoral Systems; Dehesa Ecosystems; and Veteran Rural Trees	45
Urban areas	Urban Heritage Forests, Street Trees, and Historic Habitats: An Academic Perspective	80
Special areas	Tropical Rainforests, Old-Growth Forests, Research Reserves, and Experimental Areas	60
Cross-scale and General Studies	A Comprehensive Examination of Ancient Trees: Global Perspectives, Policy Evaluation, and International Case Analyses	32

**Table 2:** Number of Publications by Ecosystem Service Type.

Service Type	Common Indicators	Number of Publications
Supporting Services	Habitat structure and provision (n = 39), biodiversity (species richness, Red List species) (n = 41), soil microbial functions (n = 16), tree growth rate (n = 14)	103
Regulating Services	Canopy shading (n = 27), temperature regulation (n = 32), microclimate mitigation (n = 25), carbon storage (n = 34), light regulation (n = 16)	79
Cultura Services	Historical and cultural value (n = 20), local identity (n = 15), spiritual symbolism (n = 13), educational value (n = 10), traditional knowledge (n = 9)	46
Provisioning Services	Forest products (n = 2), edible plants (n = 1)	3

**Table 3:** Common Methods for Different Ecosystem Service Types.

Service Type	Classification	Common Methods	Data Sources	Advantages	Disadvantages	Applicable Scales
Regulating Services	Carbon sequestration	Biomass method [45]	DBH, tree height, allometric equations	Low cost; focuses on biomass accumulation and carbon stock	Relatively low accuracy	An individual to stand on the scale
		Photosynthesis method (LI-COR) [46]	Net photosynthetic rate (Pn), leaf area	Suitable for single-tree studies focusing on physiological processes and carbon assimilation	High cost	Leaf to individual scale
		Gas chamber/flux measurement [47]	CO_2_ flux data	High precision; directly measures carbon exchange processes; suitable for mechanistic studies	Spatial Limited spatial representativeness; susceptible to disturbance; relatively high equipment cost	Individual to small-plot scale
	Microclimate regulation and thermal environment mitigation	Direct measurement [48,49]	On-site weather sensor data, including temperature, humidity, wind speed, and radiation; simultaneous observations at control and sample plots	Clear and reliable data; directly reflects the cooling and humidifying effects of ancient trees; relatively simple to operate; suitable for single-tree or small-scale studies	Limited spatial representativeness; strongly affected by short-term weather conditions; difficult to capture long-term dynamics	The individual is to stand on the scale.
		Eddy covariance [50]	High-temporal-resolution CO_2_, water vapor, and energy flux data from flux towers	Directly measures carbon, water, and energy exchange at the ecosystem scale; high accuracy; regarded as the gold standard; suitable for long-term continuous observation.	Expensive equipment; high requirements for terrain and surface homogeneity; relatively coarse spatial resolution, making it difficult to capture characteristics of individual ancient trees	Ecosystem to landscape scale
		Isotopic tracer [51]	Stable isotope data related to water or carbon cycling	Can identify water sources and carbon assimilation pathways; reveals physiological processes and environmental response mechanisms; suitable for mechanistic studies	High cost; complex experimental procedures; difficult to apply widely at large scales	An individual to stand on the scale
		Model construction [52]	Remote sensing data (for example, NDVI and LST), meteorological data, tree structural parameters, and field calibration data	Enables multiscale simulation of ecosystem services from single trees to regions; supports scenario prediction and planning applications; integrates multiple data sources.	High uncertainty; requires calibration with field measurements	Individual to regional scale
		Stem heat balance method [53]	Stem sap flow sensor data, such as heat flux and temperature gradient	Allows continuous monitoring of individual transpiration; suitable for large ancient trees; provides detailed information on water use and transpiration processes	Complex sensor installation; high equipment cost; complicated data processing	Individual scale
	Air purification	Leaf surface/bark sampling [54,55,56]	Amount of particulate matter (PM_2.5_/PM_10_) deposited per unit leaf or bark area, measured by washing, filter membrane, or swab methods	Direct and low cost; suitable for ancient tree studies	Representativeness depends on sampling location; rainfall can affect results	Individual scale
		Dry deposition model [55,57]	Leaf area, tree species characteristics, pollutant concentrations	Can estimate annual removal of PM and NO_2_/O_3_ at the kg/tree/year scale; suitable for evaluating the ecological value of ancient trees	Based on multiple assumptions and sensitive to actual deposition rates	Individual to regional scale
		Wind tunnel experiment [58,59]	PM capture efficiency of different leaf structures under controlled wind speed and particle size.	Allows analysis of microstructural effects	Does not reflect real-world conditions	Leaf to organ scale
		Dense sensor array [55,60]	PM/NO_2_/O_3_ sensor data collected at different canopy heights	High scene realism; suitable for studies of ancient tree canopy layers	Strongly affected by wind fields and footprint effects; requires more complex statistical processing	An individual to stand on the scale
Supporting Services		Field investigation [61]	Tree species composition, species records, habitat structure (tree hollows, deadwood, etc.)	Accurate data with clear ecological significance; suitable for biodiversity assessment	Time-consuming; affected by human disturbance and sample representativeness	Plot on a regional scale
		Geostatistical analysis [62]	Spatial distribution data, species location data	Reveals spatial patterns and ecological processes; suitable for multiscale analysis	High data-quality requirements; model interpretation depends on assumptions	Plot to landscape scale
		QSM/TLS scanning [63]	Three-dimensional structural data and spatial position data	Accurately captures tree structure and habitat complexity; suitable for structural characterization and multiscale analysis	High equipment and data-processing requirements; limited accessibility in some field settings	Plot to landscape scale
Provisioning Services	-	Literature review, traditional knowledge surveys	Literature sources and field survey data	Can assess the utilitarian value of timber and other biological resources	The geographical Strong geographic limitations; ecological impacts may be overlooked	Regional to social-system scale
Cultura Services	-	Literature review, semi-structured interviews, traditional knowledge documentation, policy analysis, sentiment analysis	Policy documents, historical records, and interview data	Can integrate ecological and cultural values; facilitates policy application	Strong qualitative orientation; lack of quantitative indicators; susceptible to subjectivity	Community to regional scale (social–spatial scale)

## Data Availability

No new data were created or analyzed in this study. Data sharing is not applicable to this article.

## Supplementary Materials

The following supporting information can be downloaded at: https://www.mdpi.com/article/10.3390/plants15132064/s1, File S1: The literature catalogue.
